# Interactions with Astroglia Influence the Shape of the Developing Dendritic Arbor and Restrict Dendrite Growth Independent of Promoting Synaptic Contacts

**DOI:** 10.1371/journal.pone.0169792

**Published:** 2017-01-12

**Authors:** Ginger S. Withers, Jennifer R. Farley, Jeffrey R. Sterritt, Andrés B. Crane, Christopher S. Wallace

**Affiliations:** Department of Biology, Whitman College, Walla Walla, Washington, United States of America; Centre National de la Recherche Scientifique, FRANCE

## Abstract

Astroglia play key roles in the development of neurons, ranging from regulating neuron survival to promoting synapse formation, yet basic questions remain about whether astrocytes might be involved in forming the dendritic arbor. Here, we used cultured hippocampal neurons as a simple *in vitro* model that allowed dendritic growth and geometry to be analyzed quantitatively under conditions where the extent of interactions between neurons and astrocytes varied. When astroglia were proximal to neurons, dendrites and dendritic filopodia oriented toward them, but the general presence of astroglia significantly reduced overall dendrite growth. Further, dendritic arbors in partial physical contact with astroglia developed a pronounced pattern of asymmetrical growth, because the dendrites in direct contact were significantly smaller than the portion of the arbor not in contact. Notably, thrombospondin, the astroglial factor shown previously to promote synapse formation, did not inhibit dendritic growth. Thus, while astroglia promoted the formation of presynaptic contacts onto dendrites, dendritic growth was constrained locally within a developing arbor at sites where dendrites contacted astroglia. Taken together, these observations reveal influences on spatial orientation of growth as well as influences on morphogenesis of the dendritic arbor that have not been previously identified.

## Introduction

Neighboring neurons mutually influence the shape of the dendritic fields they develop through dendrite self-avoidance [1], but nonneuronal cells like astroglia could provide spatial cues as well. *In vivo*, the onset of astrogliogenesis occurs before robust dendritic outgrowth begins [2–4]. As dendrites extend and synapses begin to form, astroglia change shape and assemble into territories with minimal overlap [5]. Notably, these events coincide, with extensive changes in the dendritic arbor occurring between 1 and 3 weeks postnatally [6], and evidence of astroglial tiling around postnatal day 14. The parallel developmental timecourse between dendritic morphogenesis and astrogliomorphogenesis suggests opportunities for regulatory crosstalk. Astroglia could secrete signals, as well as provide physical cues to guide dendrite outgrowth differentially within the arbor.

A growing body of evidence shows crosstalk between neurons and glial cells can modify the extracellular environment to influence multiple aspects of nervous system development [7–10]. For example, proteoglycans may act as morphogens in early development, but when produced by astroglia, particularly in response to injury, can be inhibitory to axonal growth [11]. There are clear examples of positive effects as well. Factors secreted by astroglia promote neuronal survival [12, 13], facilitate the onset of synapse formation [14–18], modulate synaptic efficacy [19] and regulate synapse pruning [20]. In addition, evidence suggests that exposure to astroglia can affect the developmental competence of a neuron, effectively altering rates of synapse formation [21, 22]. A number of growth factors have been identified that effect dendritic growth of forebrain neurons, e.g. [23, 24], some of which are produced or regulated by astroglia [7, 25]. Still, little is known about the ways in which exposure to astrocytes might impact the course of dendritic development.

To test the hypothesis that interactions with astroglia affect the size and shape of the dendritic arbor, we used a culture system in which the ontogeny of the dendritic arbor has been well characterized and quantified. Rat hippocampal neurons were grown in the presence or absence of astroglial cells, and the length and branching patterns of the dendritic arbor were analyzed. We found that astroglia exerted effects based on direct physical encounters, as well as paracrine influences, on dendritic growth. Astroglia constrained the extent of the dendritic arbor, even while promoting the formation of presynaptic contacts onto those dendrites. These results suggest that dendritic growth and synapse formation are not necessarily coupled and appear to involve different mechanisms. Further, the presence of astroglia generated local asymmetries in the dendritic arbor. This result suggests an influence on the orientation of dendritic processes, as well as on their growth and branching.

## Materials and Methods

### Source of tissue for primary cultures

All primary cultures were prepared from Sprague Dawley rats. All animal procedures were performed under protocols approved by the Whitman Animal Care and Use Committee, and conducted in accordance to the National Institutes of Health specifications outlined in their Guide for the Care and Use of Laboratory Animals.

### Co-plating astroglia and neurons

To analyze how physical contact between astroglia and neurons altered dendritic development, cultures were prepared by first sparsely plating astroglia onto coverslips, given time to become established, and then neurons were added to these coverslips. A detailed description of each of these steps follows.

#### Preparation of astroglial cultures on coverslips

Primary cultures of astroglia from postnatal day 1–2 rat forebrain were prepared as previously described [26], and either plated immediately or stored as frozen stocks at -80°C and subsequently revived. Astroglia preparations were plated onto poly-L-lysine-coated coverslips at low density (~ 500 cells/cm^2^), and cultured for 3 days in Minimal Essential Medium (MEM, Gibco/Life Technologies) supplemented with glucose, pyruvate, and 10% fetal bovine serum (Atlanta Biologicals). Medium was replaced after 1–2 days of glial cell plating. By 4 days *in vitro* (DIV), most were immunopositive for GFAP, a marker of differentiated astroglia, and so this timepoint was chosen to add neurons to the preparation.

#### Addition of neurons to coverslips containing astroglia

Hippocampal neurons were prepared from embryonic day 18 rats as previously described [26] and plated at low density onto the glial coverslips. Because glial cells will proliferate, visual inspection was used to confirm that the astroglia were distributed in patches separated by spaces of empty glass sufficient for some neurons to grow in isolation prior to neuron plating. Once the neurons became attached (2–3 hours), the coverslips were inverted and maintained in serum-free medium, composed of Neurobasal medium (Nb, Gibco/Life Technologies) with N2 supplements [27] in a dish with an additional physically separate astroglial feeder layer. Cytosine arabinoside (Sigma) was added 2 days after neuron plating to prevent further proliferation of glial cells. Coverslips from each experimental condition were fixed at 3–8 days after neuron plating to analyze neuron development.

### Neuron cultures with a separate astroglial feeder layer

A second set of experiments was designed to test for effects of soluble astroglial factors on dendritic growth by plating neurons and astroglia separately, with neurons grown on coverslips and astroglia on the floor of the culture dish. Briefly, low density cultures of hippocampal neurons were prepared from embryonic day 18 rats, plated onto glass coverslips (~100,000 neurons were added to a 60 mm culture dish in the initial plating) and co-cultured with a physically separate feeder layer of astroglia (~75% confluent) as described previously [26]. Cultures were grown in Neurobasal medium (Gibco/Life Technologies) with N2 [27] and glutamine or glutamax supplements. To test for the effects of glial-deprivation, some coverslips containing neurons were removed from the glial feeder layer at 3 DIV, before the onset of robust dendrite outgrowth and synaptogenesis [28, 29], then transferred to medium that had been pre-conditioned by glial cells for 12–24 hours. This relatively brief glial conditioning was sufficient for neuron survival, but unlike more extensive conditioning procedures e.g. [16], did not promote significant synaptic contact between neurons (see results below). In a subset of dishes, 40 ng/ml of human recombinant thrombospondin (TSP1, from Sigma) was added on the day of transfer (3 DIV). Coverslips from each experimental condition were fixed and analyzed 1–3 days later (at 4, 5, and 6 DIV).

### Immunocytochemistry

Cells were fixed (4% paraformaldehyde, 4% sucrose in phosphate buffered saline [PBS], pre-warmed to 37° C, 15’), followed by two brief PBS rinses [< 1 min.] and permeabilized for immunostaining (0.25% Triton X100 in PBS, 10’). See Withers and Banker [30] for detailed descriptions of immunostaining steps, but briefly, nonspecific staining was blocked using 10% bovine serum albumin (BSA) in PBS (1hr, 37°). All antibodies were diluted in 2% BSA in PBS. Primary antibody incubations were overnight at 4° C, and secondary antibody incubations were for 1 hr at 37° C, with three 5’ PBS rinses after each antibody incubation. Dendrites were identified by antibody staining to the dendritically-localized protein MAP2 (1:2000, antibody HM-2, Sigma) and anti-mouse Alexa 488 (1:400, Molecular Probes/Invitrogen); presynaptic terminals were identified using polyclonal antibodies to synapsin I (1:1500, from Millipore) and anti-rabbit Cy3 (1:400, Jackson Immunolabs). In some cases, neurons were also identified using neuron-specific tubulin (monoclonal Tu20, from AbCam, 1:500). Astroglia were identified using antibodies to GFAP (monoclonal, Sigma; polyclonal, Santa Cruz) and the same anti-mouse and anti-rabbit secondary antibodies as above, or visualized using rhodamine phalloidin (Cytoskeleton). Coverslips were mounted onto slides with an aqueous polyvinyl mounting medium as per Withers and Banker (1998; Elvanol, DuPont) containing an anti-bleaching agent (Dabco, Sigma-Aldrich).

### Scanning electron microscopy

Cells were prepared as according to [31] using a 4% glutaraldehyde solution in PBS, and then transitioned through a 10–100% graded ethanol dehydration series. After dehydration, cells were dried using critical point drying methodology [32]. Lastly, cells were coated with Gold or Gold–palladium with either a Cressington Model 108 Sputter Coater (Whatford, England) or a PELCO Model 3 Sputter Coater 91000 (Ted Pella, Redding, CA). Digital images of cells were acquired with an FEI Quanta 250 electron microscope.

### Fluorescence microscopy and quantification

Images were acquired using a Leica IRB microscope with a Prior motorized stage, controlled with MetaMorph software, and a Photometrics CoolSnap CCD camera, or a Leica SP5 confocal microscope. For quantification, immunofluorescent images from each experimental condition were acquired across 2–3 independently prepared cultures (15–20 digital images per culture replication, 0.168μm/pixel) with a 40X Fluotar objective using a forced sampling scheme (moving in the X and Y axes at predetermined intervals to sample from the entire coverslip). Bandpass filters for optimized for GFP and Rhodamine wavelengths (Chroma) ensured that independent capture of fluorescent signals with no overlap. Images acquired using the SP5 were gathered using sequential scans, with wavelength capture settings optimized to exclude non-target fluorphores. Exposure times were kept constant across experimental conditions for each fluorophore. If no cells were present in the assigned position, the stage advanced to the next position. If cells were present, but partially out of frame, the stage was repositioned to move the neuron such that the dendritic arbor could be completely contained within the frame. In the event that an entire arbor was larger than could be contained within the frame, an additional image was acquired using a 20X Fluotar objective, enabling quantification of the full extent of the arbor. Most images acquired had only a single neuron present in the frame, but when more than one neuron was present, measurements were made from all neurons that had dendritic arbors contained completely within the frame, provided that they could be clearly distinguished from neighboring neurons. Digital files were coded and, whenever possible, analyzed blind to experimental condition (i.e. sometimes presence of glial cells was unavoidably obvious). Double- and triple-stained images were combined to yield a merged image to ensure accuracy in analysis of neuron-glia spatial relationships, and confirm that only puncta in contact with MAP-2 positive dendrites were counted. Neurons were classified based on their relationship with astroglia (full contact, partial contact, no contact), and quantified using Image J functions. Dendrite length was estimated using a Sholl concentric ring analysis, with rings placed at 10 μm intervals [33].

**Fig 1:**
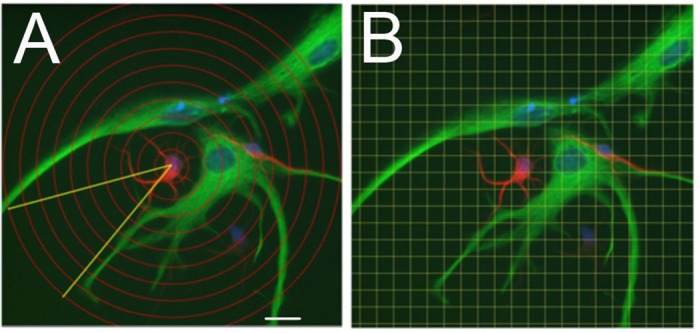
Scheme for quantifying bias in the pattern of dendritic growth with respect to the location of astroglia. **A)** To measure the radial extent of the dendritic arbor, concentric Sholl rings were placed around the neuronal cell body and repeated at 10 μm intervals. To provide an index of the potential growth zone occupied by glia, the 360° Sholl area was divided into a sector occupied by glia and a sector absent of glia indicated by the angle shown in yellow. **B)** The fraction of the Sholl area occupied by glia was measured by superimposing a 10 μm x 10 μm grid onto the image and counting intersections. Scale bar = 20 μm.

The extent to which dendrites and filopodia oriented toward nearby astroglia was also measured. To quantify the pattern of dendrite arborization in relation to the astroglial cell, a zone of glial proximity was determined using a “pizza wedge analysis,” defined by placing the vertex of an angle at the center of the neuronal cell body (Fig 1). The rays of the angle were extended to the two farthest boundaries of the nearby astroglial cell(s). Concentric rings were superimposed at 10 μm intervals around the neuronal cell body, and the number of intersections by dendrites falling within, and outside of, the wedge was counted. The area covered by glia within a 100μm radius of the neuronal cell body was estimated using the ImageJ Grid Overlay plugin. Grid squares (10μm x 10μm) were superimposed over the image, and the area of glia within each Sholl ring was estimated to the nearest half-square. To determine filopodial density, the length of a process segment was measured using ImageJ, and the number of filopodia along that length were counted and divided into 2 categories based on orientation towards or away from glia.

In statistical analyses, the distribution of each experimental condition was tested using the D’Agostino and Pearson omnibus normality test (Graphpad Prism). In cases of normal distribution, a Student’s t-test, or an ANOVA with posthoc Tukey comparisons, was used to determine significant differences between multiple groups (JMP software, or Graphpad Prism). If any group deviated from a Gaussian distribution, then a nonparametric test, either Wilcoxon’s matched pairs, or Kruskal-Wallis with Dunn’s multiple comparison test, was applied using Graphpad Prism.

## Results

### Contact with astroglia constrained dendritic growth

**Fig 2:**
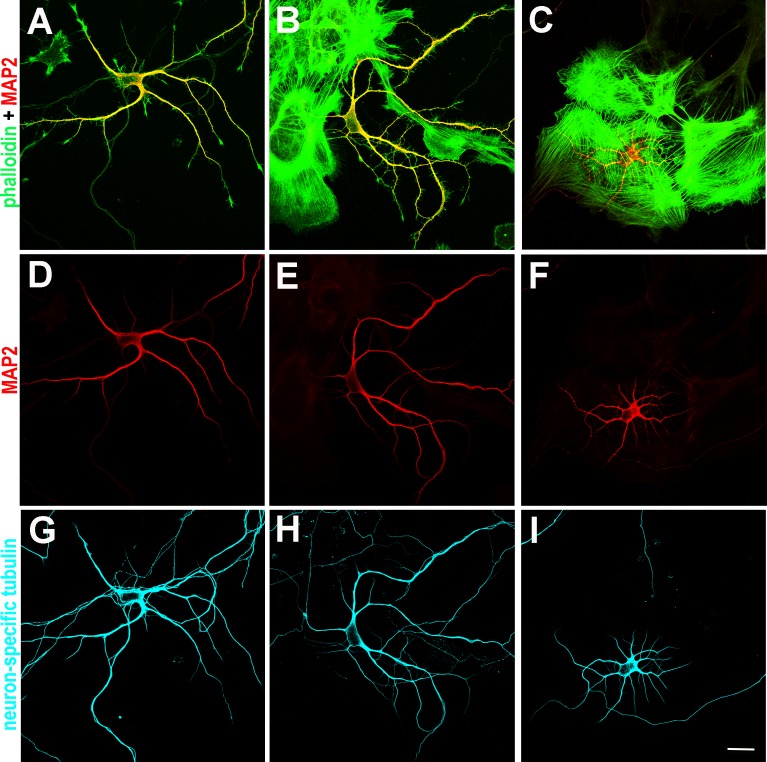
Plating neurons and astroglia together at low density produced varied degrees of interaction between the two cell types. Cells were stained to reveal the dendritic arbor of neurons (MAP2, red) and extent of interaction with astroglia. Phalloidin (green) binds filamentous actin, is present throughout astroglia, as well as neurons, and is notably concentrated in growth cones. Combining these two fluorescent channels distinguished the boundaries and overlap between the two cell types (as in A, B, and C). Comparing MAP2 staining (D, E, F) against neuron-specific tubulin (G, H, I) allowed dendrites to be distinguished from axons and further separated neurons from astroglia. Each column shows representative neurons in differing degrees of interaction with astroglia: neurons with dendritic arbors growing without physically contacting astroglia (A, D, G); arbors in partial contact with astroglia (B, E, H); and arbors in full contact with astroglia (C, F, I). Scale bar = 20 μm.

**Fig 3:**
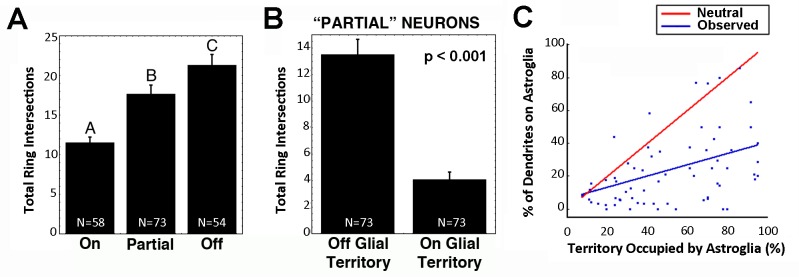
Contact with astroglia produced asymmetric dendritic arbors through local restriction of growth. **A)** The total number of Sholl ring intersections for dendrites growing completely on (On), partially on (Partial), or not in contact (Off), showed that dendritic growth was inversely proportional to the extent of physical contact with astroglia. Bars with different letters were significantly different from each other. **B)** For neurons in partial contact, the number of dendrites intersecting Sholl rings was significantly reduced for the portion of the arbor in contact with astroglia (On Astroglial Territory) compared with the portion that was not (Off Astroglial Territory). **C)** Scatter plot showing localized limited dendritic growth based on physical contact with astroglia. If the presence of glia had no effect on growth, the probability of dendrites falling on glia would simply be a function of the proportion of the field occupied by glia (“neutral,” red line). The slope of the line of best fit to data (“observed,” blue line) falls below the neutral line, indicating that dendritic growth was restricted when it entered the territory of an astroglial cell. Neurons were quantified at 4 DIV. Data are reported as mean +/- standard error (SE).

To test whether interactions with astroglia altered radial expansion of dendrites, neurons were plated onto coverslips that contained a sparse distribution of astroglial cells. This created a sample with varied degrees of interaction between the astroglia and the neuron (Fig 2). All neurons formed a dendritic arbor, but the pattern of outgrowth appeared to depend on the amount of contact. Dendrites of neurons not in contact with astroglia were relatively long, whereas dendrites growing on astroglia were shorter. Quantification supported these observations (Fig 3A, one-way ANOVA, F = 11.79, p < 0.0001 with Tukey post hoc comparisons, α = 0.05). The dendrites of neurons in full contact with astroglia had significantly fewer ring intersections than those with no contact. Neurons that were in partial contact had an intermediate number of intersections and were significantly different from both those in full, and those without, astroglial contact.

### Astroglia contact created local asymmetry in dendritic arbors

Neurons in partial contact with astroglia provided an opportunity to test whether the reduced arbor size was a cell-wide general inhibition of growth. If astroglia inhibited the growth of dendrites in a general manner, then a neuron in partial contact would be expected to have an arbor that is reduced uniformly throughout its extent. Conversely, a mechanism dependent on local interactions would produce a lop-sided arbor, with shorter dendrites in physical contact with glia, and longer dendrites in areas not under physical influence. Analyses that separated regions of the arbor based on position relative to astroglia showed that partial contact with astroglia did indeed lead to significant asymmetry in the dendritic arbor (Fig 3B). The portion of the arbor in physical contact was decreased significantly compared to the portion not in contact (Wilcoxon matched pairs signed rank test, two-tailed, W = 1768, p < 0.001, Spearman rs = -0.28).

Simply tallying the number of rings intersected by parts of the arbor does not take into account the fraction of the area that is filled by islands of astroglia, however. Thus, we tested the null hypothesis that the amount of dendrite in contact with astroglia simply reflected the amount of territory surrounding the neuron that was occupied by astroglia. Fig 3C represents the percent of the field occupied by astroglia plotted against the percent of the dendritic arbor that was in contact with the astroglia. The “neutral” line shown predicts the relationship between astroglia and dendrite arborization if they were proportionately scaled, i.e. if 25% of the territory surrounding the neuron was occupied by astroglia, then 25% of the arbor would be expected to occupy that territory as well. If astroglia were strongly attractive, then one would expect most data points to fall above this line, if interactions with astroglia restricted growth, then most points would fall below the line. We found that over 90% of the cases were below the neutral line, strongly supporting the hypothesis that local interactions with astroglia limited dendritic growth.

### Evidence dendrites grow toward nearby astroglia

**Fig 4:**
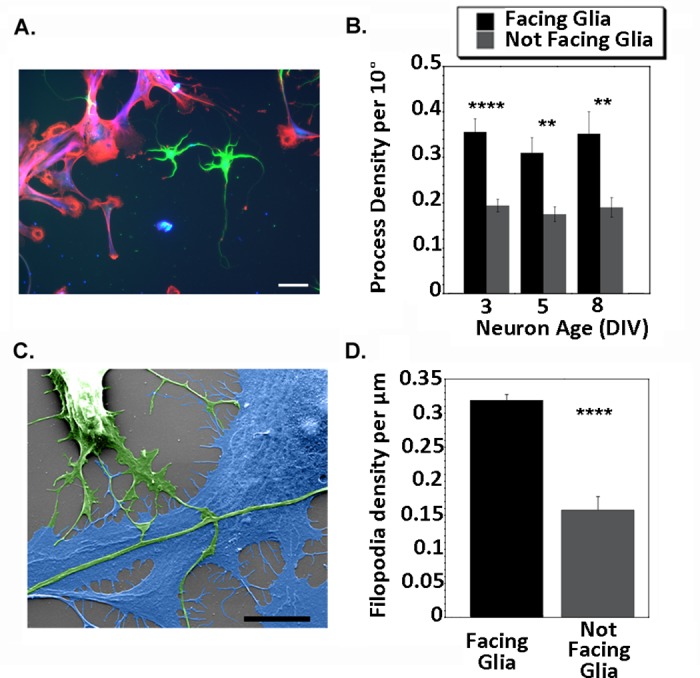
Dendrites and filopodia orient toward astroglia. **A)** Representative image of neuronal dendrites at 3 DIV (green, MAP2 staining) plated with astroglia (red/blue, phalloidin/GFAP), scale bar = 50 μm. **B)** Quantification. Dendritic arbors of neurons at 3, 5 and 8 DIV were divided into zones based on the relative position of astroglia. The dendrites facing vs. not facing astroglia were counted using a pizza-wedge analysis, as described in Methods (see Fig 1). To correct for varying amounts of territory occupied by astroglia in the field, the number of dendrites per 10° is reported. **C)** Representative SEM image of a neuron and neighboring astroglia at 3 DIV, false-colored to highlight processes and filopodia of both cell types. Scale bar = 10 μm. **D)** Mean number of filopodia per micron of neuronal process that oriented toward vs. away from nearby astroglia. For panels B and D, data are reported as means +/- SE; each DIV was analyzed independently using a paired, two tailed test as described in results. ****, p < 0.0001; **, p < 0.01.

Although direct contact with astroglia restricted dendritic growth, we found evidence that, when near to, but not in contact with the glial cell, dendrites showed a directional bias toward the astroglia (Fig 4A and 4B). Images that contained non-overlapping neurons and astroglia were analyzed by dividing the area surrounding the neuron into glial-facing and non-glial-facing zones. The number of dendrites that fell within and outside of the facing zone were counted (as shown in Fig 1). Neurons analyzed 3–8 DIV following plating all showed a similar—and significant—orientation bias, with dendrites radiating toward astroglia (3 DIV, n = 20 neurons, t = 6.05, p<0.0001; 5 DIV, n = 10 neurons, t = 4.63, p = 0.001; 8 DIV, n = 10 neurons, W = -53, p = 0.004).

**Fig 5:**
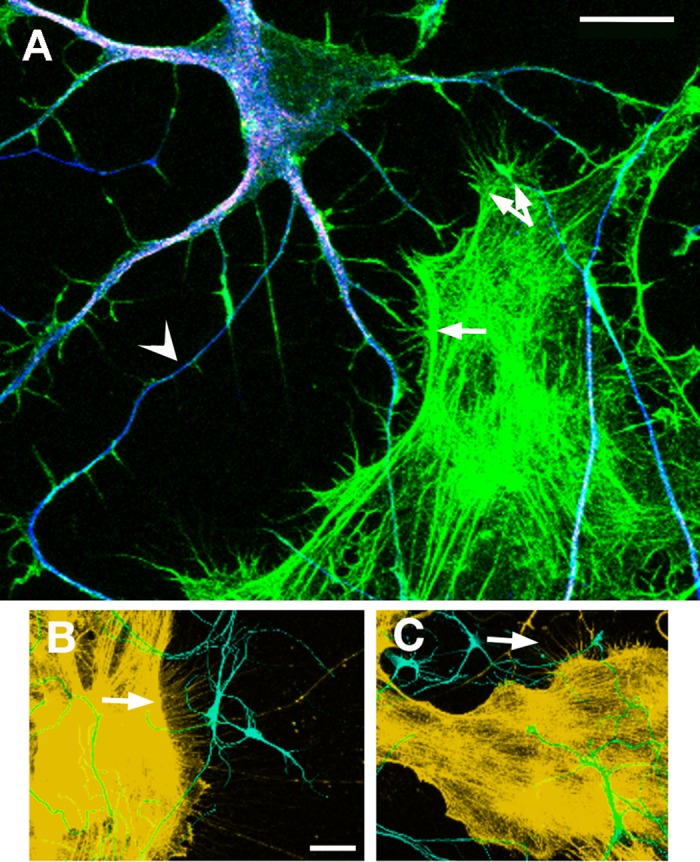
Astroglia and dendrites interact via filopodial contact. Phalloidin staining of filamentous actin reveals extensive filopodia on both neurons (shown at 5 DIV) and astroglia. **A)** High magnification image shows numerous zones of astroglial filopodia (arrows), resulting in frequent contact between filopodia of both cell types. The combined image shows filamentous actin (phalloidin staining, green), MAP2 staining (pink), and neuron-specific tubulin (blue), arrowhead points to the axon, scale bar = 20 μm. **B, C)** Images acquired using lower magnification show patches of astroglia with numerous, long filopodia (arrows, orange) extending toward neurons stained with neuron-specific tubulin (teal), to reveal all processes (including both axons and dendrites). Scale bar for B, C = 50 μm.

Actin-rich filopodia are commonly thought to act as sensors of the local extracellular environment and provide guidance cues during outgrowth. To test whether the distribution of neuronal filopodia were also biased toward astroglia, images were gathered using SEM, which offered higher resolution than conventional light microscopy. Similar to the biased orientation of dendrites, significantly more neuronal filopodia were oriented toward glia rather than away (Fig 4C and 4D, 3 DIV, n = 18 segments of dendrites from neurons at 3 DIV, W = -169.0, p < 0.0001). Together, these data could suggest a chemoattractive gradient by which astroglia release diffusible signals toward which both dendrites and filopodia radiate. Fig 5 suggests an alternative physical mechanism. In addition to neuronal filopodia, phalloidin staining revealed thin filopodial extensions streaming from astroglia towards neurons (see also Fig 2B), and these extensions could be quite long (see Fig 5B, where arrows highlight astroglia filopodia that extend well beyond 50 μm in length). We did not test whether astroglial filopodia were biased to dendrites, but they were also observed radiating toward axons. These filopodial interactions could produce transient physical contact initiated by either cell type that guide, or even pull, dendrites toward astroglia.

### Astroglial paracrine influences are inhibitory to dendritic growth, but promote formation of presynaptic contacts

It is already well established that astroglia secrete factors that can promote at least some stages of synapse formation [16, 17]. We sought to determine whether secreted factors also influenced dendritic growth, as well as to replicate synaptogenic effects. Immature dendrites (also called minor processes) form during the first 24–48 hours *in vitro*, but robust dendritic growth and synaptogenesis begin around 3 DIV. Therefore, we cultured neurons in the presence of an astroglial feeder layer until 3 DIV, at which time we transferred the coverslips containing neurons into dishes minus the feeder layer, but with medium that had been conditioned only briefly (less than 24 hours). Previously, brief conditioning has helped to maintain neuron survival, but as shown here, it was not sufficient to promote synapse formation (see below). Because the glial feeder layer was removed from the dish, this condition is described as “glial deprivation”.

**Fig 6:**
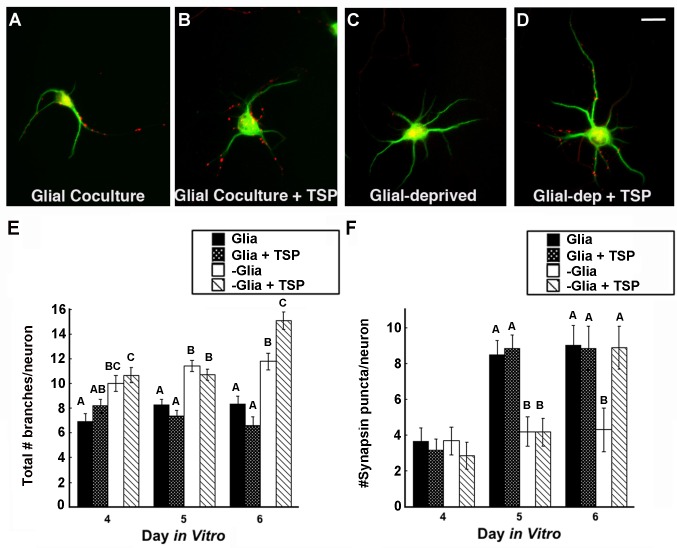
Soluble astrocytic factors modulate dendritic growth as well as synapse formation. **A—D)** Neurons at 5 DIV, immunostained for MAP2 (green) and synapsin I (red) have few presynaptic contacts when placed under glial deprivation, but more elaborate dendritic arbors. Scale bar = 15 μm. **E)** The total number of branches per neuron was counted and averaged per experimental condition. Significant differences in the total number of branches between glial deprived and +TSP conditions suggest additional factors beyond TSP are likely involved. **F)** The total number of presynaptic contacts per neuron was counted and averaged per experimental condition. For both Panels E and F, the sample size (N) of each condition at 4 DIV was 41, 60, 39, 42, from 2 separate culture preparations; at 5 DIV, 114, 112, 107, 110, from 3 separate culture preparations; at 6 DIV, 58, 50, 49, 43, from 2 separate culture preparations, respectively. Data are reported as mean +/- SE. For each age group, bars with different letters are significantly different from each other at p < 0.05 as determined by a Kruskal-Wallis test combined with Dunn’s Multiple Comparisons Test. See results for additional details of statistics.

When growing under glial deprivation, the dendritic arbors of neurons were significantly increased compared to those grown with astroglia present in the culture dish (Fig 6). Whereas glial deprivation resulted in increased dendritic arbor size, the number of presynaptic contacts on neurons was significantly reduced compared to those growing with astroglia, as previously reported. Thrombospondin (TSP) is one glial-derived secreted factor that promotes synapse formation [16], and so we also tested for effects of TSP. Neurons from deprived cultures supplemented with TSP had significantly more presynaptic contacts compared with untreated deprived neurons, with significant differences detected by 6 DIV, 72 hours after the addition of TSP to the culture (see panel F, Fig 6). Finally, adding TSP to neurons with an astroglial feeder layer did not further enhance the formation of presynaptic contacts, suggesting that the astroglial factors that promote synapse formation were already present in excess in the culture dish. Similarly, the distribution of presynaptic contacts on neurons in partial contact with astroglia did not appear differ significantly across the arbor, adding support to the hypothesis that the primary contributing factor in promoting the initial presynaptic contact is secreted rather than contact-mediated (see S1 Fig).

Significant differences in total number of branches and synapses per neuron shown in Fig 6 were determined using a Kruskal-Wallis (K-W) test combined with Dunn’s Multiple Comparisons Test. For total number of branches, K-W statistical values obtained were: 4 DIV, 18.64, p< 0.0003; 5 DIV, 48.07, p<0.0001; 6 DIV, 69.01, p < 0.0001. For total number of synapses, K-W statistical values obtained were: 4 DIV, 2.55, p = 0.47; 5 DIV, 62.65, p<0.0001; 6 DIV, 12.06, p < 0.01.

Analysis of dendrites after only 24 hours of glial deprivation showed that the total number of branches was significantly increased compared to neurons continuously maintained with an astroglial feeder layer (see neurons at 4 DIV, Fig 6E). The magnitude of those differences continued to increase over the next 48 hours (see neurons at 5 and 6 DIV, Fig 6E). Glial deprived neurons treated with TSP had significantly more branches, and increased arbor size, as well as significantly more presynaptic contacts compared with neurons from the glial deprived condition. Thus, the number of synapses on a dendritic arbor was not simply correlated with the arbor size in these experiments.

**Fig 7:**
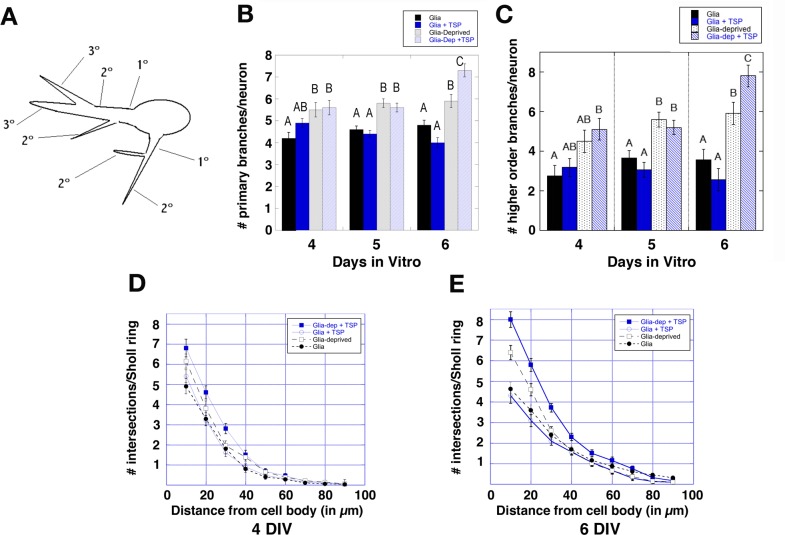
Evidence that paracrine effects act generally throughout the dendritic arbor. **A)** Nomenclature for order of branches. Primary branches emerge from the cell body, higher order branches are the sum of all branches above primary. **B)** Number of both primary, and **C)** higher order branches were increased significantly in neurons deprived of astroglia, with significant differences detected at 4 DIV (24 hours after removal of astroglia from the culture). Bars with different letters are significantly different from each other at p < 0.05. **D, E)** Sholl ring analyses corroborated branch analyses, with mean number of intersections for neurons under glial deprivation (glia-dep and glia-dep + TSP, both numerically higher at 4 DIV (D), and significantly higher at 6 DIV (E). When taken with data shown in Fig 3, these data suggest that astroglia produce general as well as contact-mediated effects on dendritic growth, potentially involving different factors. Data are reported as mean +/- SE, N is identical to that reported in Fig 6.

More fine-grained analyses tested whether the paracrine effects on dendrites were localized to proximal vs. distal regions of the arbor, or were uniformly distributed throughout the arbor (Fig 7). Both Sholl ring and branch analyses detected effects throughout the arbor, with glial deprivation resulting in significant increases in both primary and higher order branches. For analysis of primary branch number, K-W statistical values obtained were: 4 DIV, 14.23, p = 0.0016; 5 DIV, 36.77, p<0.0001; 6 DIV, 51.71, p < 0.0001. For analysis of higher order branches, K-W statistical values obtained were: 4 DIV, 10.13, p = 0.0175; 5 DIV, 29.04, p<0.0001; 6 DIV, 52.06, p < 0.0001. For analysis of Sholl rings, the total number of ring intersections was summed and compared between groups; K-W statistical values obtained were: 4 DIV, 18.83, p < 0.0003; 6 DIV, 30.55, p < 0.0001, 5 DIV was intermediate (not shown). While the contact-mediated reduction in the dendritic arbor shown in Figs 2 and 3 was quite localized to the portion of the arbor in direct contact with an astroglial cell, these data provide evidence of paracrine influences that act generally on all dendrites. Thus, it is possible that the contact-dependent effects could be due to a separate cohort of signals.

## Discussion

The purpose of this study was to characterize the influence astroglia could have on the morphogenesis of the dendritic arbor. The main findings were that: 1) Dendrites orientated toward nearby astroglia, but their growth was locally restricted upon coming into physical contact with those glia. Together, these influences generated significant asymmetry in the dendritic arbor. 2) Even in the absence of positional cues provided by neighboring cells, the general presence of astroglia in the culture environment caused a significant reduction in dendritic arbor size as compared with dendritic arbors of neurons growing under glial deprivation. This was observed regardless of whether the neurons were plated onto the same coverslip as astroglia, or were prepared with a physically separate feeder layer. These findings offer evidence that astroglia influence multiple aspects of dendritic growth and patterning, and that the regulatory interactions involve a combination of paracrine and contact-mediated signals derived from astroglia.

**Fig 8:**
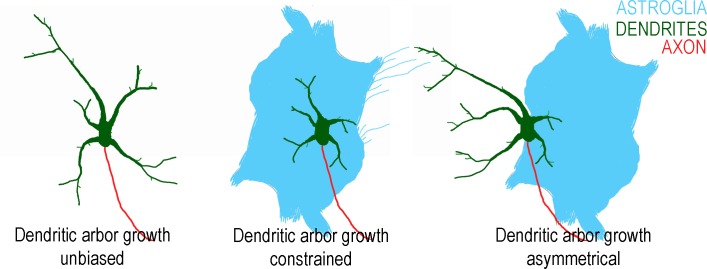
Local and physical interactions between dendrites and astroglia can alter the shape and growth of the dendritic arbor. Dendrites not in contact with astroglia are significantly longer than those in contact. Dendrites, and associated filopodia that are in close proximity to astroglia orient toward the glial cell which, upon contact, restricts growth.

These data demonstrate the potential importance of spatial context in interactions between astroglia and neurons (Fig 8). When neurons were nearby astroglia, filopodia extended from both cell types toward each other, and dendrites oriented toward the glial cell. Upon contact, however, the growth of the dendritic arbor of a neuron became asymmetric, with short dendrites in contact with the glial cell, and longer ones directed away. Although we did not quantify axons in this study, they did not appear to show the same kinds of contact-dependent constraint (see for example, Fig 5B and 5C, where neuron-specific tubulin staining shows axons traversing across patches of glia).

Astroglia are capable of producing signals that are inductive, as well as inhibitory, to neuron development [34]. It may be that attraction of dendrites toward astroglia and restriction of dendrite growth upon contact are the product of interactions analogous to the set of stages shown to be involved in regulating the building, maintenance, and elimination of synapses [35, 36]. Alternatively, a simple, adhesive spatial capture mechanism could account for these findings. For example, filopodia from both cell types could extend stochastically, and upon encountering each other, adhere and then pull dendrites toward the astroglia. Once there, adhesive interactions could limit dendrite outgrowth. There are several candidate adhesion molecules that could mediate this kind of effect [37, 38].

The experiments described here were designed to distinguish influences involving localized physical contact between growing dendritic branches and astroglia from influences that simply required astroglia to be present in the same dish. Generally, astroglia inhibited growth of dendrites, evidenced by decreased numbers of branches, and length across the entire arbor. Not surprisingly, based on previous findings [16], when astroglia were removed from the culture, few presynaptic contacts formed. Meanwhile, the size of the dendritic arbor increased significantly. This suggests that synapse formation and dendrite growth can be uncoupled. This dissociation provides counterpoint to the “synaptotrophic hypothesis” which proposes that dendritic outgrowth and synaptogenesis are inter-dependent [39, 40].

Some synaptogenic properties of astroglia are mediated by the soluble factor TSP [16], and so we tested whether TSP could substitute for astroglia by inhibiting dendrite growth as well. As would be expected based on previous findings, the addition of TSP to glial deprived cultures did indeed increase the number of presynaptic contacts on neurons significantly. It did not, however, mimic astroglial inhibition of dendritic development. Thus, these experiments exclude TSP as a candidate factor mediating this effect.

Within intact neuropil, the timeline for elaboration of dendritic arbors coincides with the timing for when astroglia establish nonoverlapping territories and take on a mature phenotype [4, 5, 41–43]. In our model, neurons were prepared from embryonic brains, whereas astroglia were obtained from postnatal preparations to take advantage of the time windows when these cell types are being generated, and are amenable to *in vitro* culturing. Not surprisingly, the timing of encounters between cell types, and the genes that are being expressed at that time, are critical in guiding development. As the influence of astroglia upon neuronal development has been examined in increasing detail, evidence has emerged showing crosstalk between neurons and astroglia can influence the developmental state of the astroglia [44–46]. In this regard, we recognize the need to be cautious in interpreting these data in the context of the intact brain. Indeed, cultured astroglia retain some patterns of gene expression similar to an immature, transitional, or reactive phenotype [47]. Nonetheless, the data presented here provide direct evidence that the spatial organization of dendrites, and the formation of presynaptic contacts on them, can be significantly influenced by astroglia.

Dendrite orientation toward, and restricted growth upon contact with astroglia provides evidence that the tessellation of astroglia across dendritic arbors may involve a more active patterning of dendritic size and branching by astroglia than previously thought. This hypothesis is supported indirectly by *in vivo* studies using two different genetic models of neurological disorders, Fragile X mental retardation and Rett syndrome. In those reports, astroglia exerted some control over the size of the dendritic arbor, an effect that was compromised by the gene defects underlying the disorders [48, 49]. Further, astroglia are thought to contribute to the construction and function of cortical circuits and maps by physically defining and coordinating synaptic territories [50–52]. Astroglial control over available postsynaptic territory could be an important part of this mechanism. Given the protracted nature of dendritic development associated with emergence of complex cognitive functions in the mammalian telencephalon, such regulatory influence might be profound.

## Conclusions

This *in vitro* model revealed that a number of morphological features of a developing dendritic arbor could be modified significantly by the presence of astrocytes, including the orientation of filopodia and dendritic branches, the extent of dendritic outgrowth, and locally restricted outgrowth based on contact with an individual astrocyte. Some effects, such as reductions in dendritic branching and overall arbor size, were the result of paracrine factors, and occurred generally throughout the arbor. Other effects, e.g. biased orientation of filopodia and dendrites toward nearby astroglia, and asymmetric growth of the arbor, occurred through local and contact-mediated interactions (Fig 8). The glial-derived synaptogenic factor thrombospondin (TSP) did not mimic these effects, but did promote the formation of presynaptic contacts, providing evidence that the mechanisms that restrict growth of the dendritic arbor are distinct from those invoked by TSP. Collectively, these results demonstrate that interactions with astrocytes can have a significant impact on the phenotype of a dendritic arbor and raise questions about how interplay between developing neurons and astrocytes might shape the dendritic arbor *in vivo*.

## Supporting Information

S1 Fig Synapsin puncta formed on dendrites both on and off of astroglia islands without significant bias. **A—C)** Neurons at 5 DIV, coplated with astroglia, stained with phalloidin (yellow) to reveal polymerized actin, and immunostained for MAP2 (red), synapsin I (green), neuron-specific tubulin (blue). Presynaptic puncta are evident both along dendrites contained within the astroglial island, and on dendrites not in direct contact with astroglia. **B)** Arrows indicate representative puncta along dendrites contained within, and outside of, the astroglial island. The boundary of the astroglial island is identified by white outline. Scale bar, 25μm. **D)** Quantification of puncta per micron showed a nonsignificant, modestly higher density along dendrites in contact with astroglia. Only puncta along side dendrites labeled with MAP2 staining were counted. Dendrites from 22 neurons in partial contact were analyzed using a paired two-tailed t-test, p = 0.21. Synapse density for 32 neurons from a Banker-style co-culture (i.e. neurons were physically separate from a monolayer of astroglia) using the same neuron preparation yielded a mean density of 0.065 +/- 0.009 (SE) contacts per micron. While astroglia have been shown to play a significant role in regulating synapse formation through diffusible factors, e.g. (Christopherson et al., 2005), the data shown here suggest that physical contact with astroglia does not exert a dominant influence over the location where initial presynaptic contacts form along a dendrite. It is clear, however, that the assembly of functional synapses is a multistep process and that glia signals appear to be more influential in some stages than others (Stevens, 2008). These data, therefore, may be limited in that they assess the localization of a single presynaptic marker, Synapsin I, during one specific developmental stage of synaptic assembly.
(PDF)
