# Supplementary material for: Interactions with Astroglia Influence the Shape of the Developing Dendritic Arbor and Restrict Dendrite Growth Independent of Promoting Synaptic Contacts
Source: PLoS One. 2017 Jan 12;12(1):e0169792. doi: 10.1371/journal.pone.0169792 (PMC5233417; doi:10.1371/journal.pone.0169792)

S1 Fig. Synapsin puncta formed on dendrites both on and off of astroglia islands without significant bias. A t   C) Neurons at 5 DIV, coplated with astroglia, stained with phalloidin (yellow) to reveal polymerized actin, and immunostained for MAP2 (red), synapsin I (green), neuron-specific tubulin (blue). Presynaptic puncta are evident both along dendrites contained within the astroglial island, and on dendrites not in direct contact with astroglia. **B)** Arrows indicate representative puncta along dendrites contained within, and outside of, the astroglial island. The boundary of the astroglial island is identified by white outline. Scale bar, 25µm. **D)** Quantification of puncta per micron showed a nonsignificant, modestly higher density along dendrites in contact with astroglia. Only puncta along side dendrites labeled with MAP2 staining were counted. Dendrites from 22 neurons in partial contact were analyzed using a paired two-tailed t-test, $p = 0.21$. Synapse density for 32 neurons from a Banker-style co-culture (i.e. neurons were physically separate from a monolayer of astroglia) using the same neuron preparation yielded a mean density of 0.065 +/- 0.009 (SE) contacts per micron. While astroglia have been shown to play a significant role in regulating synapse formation through diffusible factors, e.g. (Christopherson et al., 2005), the data shown here suggest that physical contact with astroglia does not exert a dominant influence over the location where initial presynaptic contacts form along a dendrite. It is clear, however, that the assembly of functional synapses is a multistep process and that glia signals appear to be more influential in some stages than others (Stevens, 2008). These data, therefore, may be limited in that they assess the localization of a single presynaptic marker, Synapsin I, during one specific developmental stage of synaptic assembly.

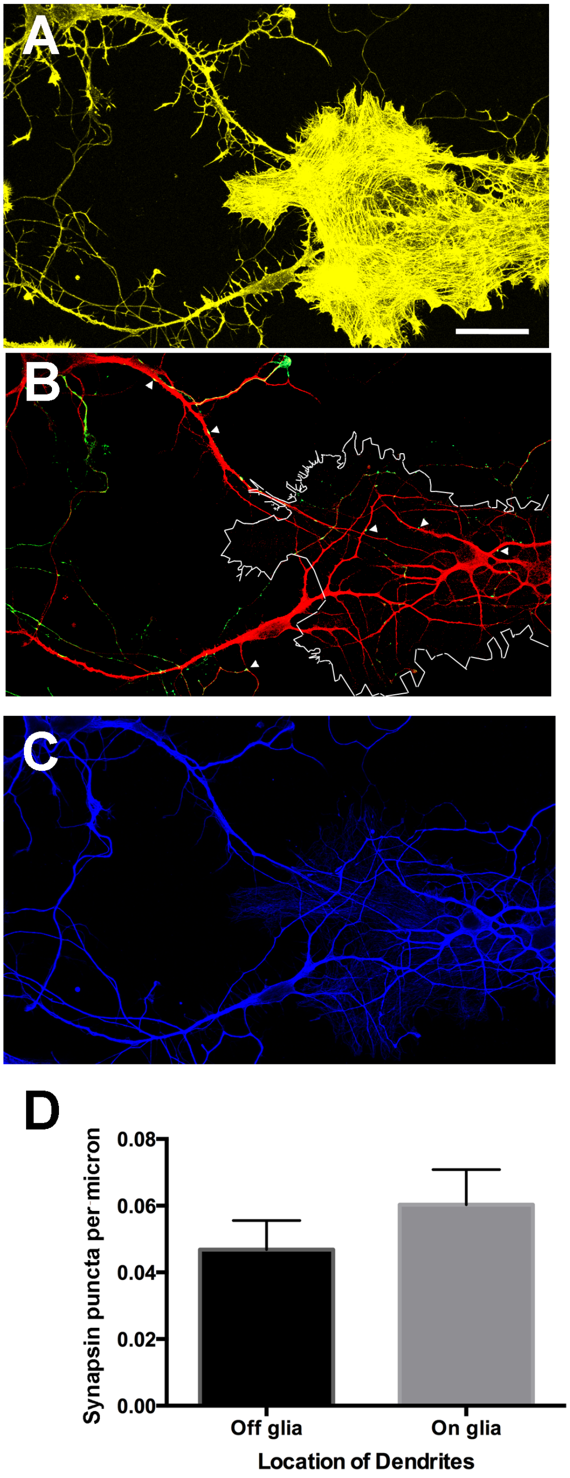

Supplement: S1 Fig — A—C) Neurons at 5 DIV, coplated with astroglia, stained with phalloidin (yellow) to reveal polymerized actin, and immunostained for MAP2 (red), synapsin I (green), neuron-specific tubulin (blue). Presynaptic puncta are evident both along dendrites contained within the astroglial island, and on dendrites not in direct contact with astroglia. B) Arrows indicate representative puncta along dendrites contained within, and outside of, the astroglial island. The boundary of the astroglial island is identified by white outline. Scale bar, 25μm. D) Quantification of puncta per micron showed a nonsignificant, modestly higher density along dendrites in contact with astroglia. Only puncta along side dendrites labeled with MAP2 staining were counted. Dendrites from 22 neurons in partial contact were analyzed using a paired two-tailed t-test, p = 0.21. Synapse density for 32 neurons from a Banker-style co-culture (i.e. neurons were physically separate from a monolayer of astroglia) using the same neuron preparation yielded a mean density of 0.065 +/- 0.009 (SE) contacts per micron. While astroglia have been shown to play a significant role in regulating synapse formation through diffusible factors, e.g. (Christopherson et al., 2005), the data shown here suggest that physical contact with astroglia does not exert a dominant influence over the location where initial presynaptic contacts form along a dendrite. It is clear, however, that the assembly of functional synapses is a multistep process and that glia signals appear to be more influential in some stages than others (Stevens, 2008). These data, therefore, may be limited in that they assess the localization of a single presynaptic marker, Synapsin I, during one specific developmental stage of synaptic assembly. (PDF) [file pone.0169792.s001.pdf]
